# Spatial and temporal variations in Spain in the standardised ratio of in-hospital mortality due to colorectal cancer, 2008–2014

**DOI:** 10.1186/s12885-019-5502-y

**Published:** 2019-04-03

**Authors:** J. M. García-Torrecillas, M. C. Olvera-Porcel, M. Ferrer-Márquez, F. Rubio-Gil, M J. Sánchez, M. Rodríguez-Barranco

**Affiliations:** 1 Hospital Universitario Torrecárdenas, Almería, Spain; 2 CIBER de Epidemiología y Salud Pública (CIBERESP), Madrid, Spain; 3 Fundación FIBAO, Hospital Universitario Torrecárdenas, Almería, Spain; 4 Escuela Andaluza de Salud Pública, Instituto de Investigación Biosanitaria ibs, Granada, Spain; 5 Hospitales Universitarios de Granada/Universidad de Granada, Granada, Spain

**Keywords:** Colorectal cancer, Mortality, Standardised mortality ratio, Epidemiology, Trends

## Abstract

**Background:**

Colorectal cancer (CRC) is the second cause of tumour mortality in Spain and Europe. To date, no studies have been conducted in Spain to evaluate the spatial and temporal distribution of the excess risk of death during hospitalisation for CRC.

**Methods:**

A cohort was constructed of all episodes of hospitalisation in Spain due to CRC (codes 153 and 154 of the International Classification of Diseases, 9th edition, Clinical Modification) during the period 2008–2014, based on the minimum basic data set published by the Ministry of Health. Mortality ratios were calculated per region for each of the years analyzed (spatial or cross-sectional analysis) and during the overall study period, for each region independently (temporal or longitudinal analysis). In the first of these analyses, particular note was taken of the regions and years in which the limits of two and three standard deviations were exceeded.

**Results:**

Two hundred and fifty eight thousand, nine hundred and twenty seven episodes of CRC were analysed. The patients were predominantly male (60.6%), with an average hospital stay of 13.16 days. Half underwent surgery during admission and on average presented more than six diagnoses at discharge. The spatial analysis revealed mortality ratios that deviated by at least three standard deviations in the following regions: Islas Canarias, Asturias, Valencia, Extremadura, País Vasco and Andalucía. The longitudinal analysis showed that most regions presented one or more years when CRC mortality was at least 15% higher than expected during the period; outstanding in this respect were Asturias, Navarra and La Rioja, where this excess risk was detected in at least 2 years.

**Conclusions:**

Geographic and temporal patterns of the distribution of the excess risk of mortality from CRC in Spain are described using SMRs. We conclude that during the study period, the geographic pattern of mortality in Spain did not coincide with the excess risk of mortality calculated using the SMR method described by Jarman and Foster. This method of risk estimation can be a useful tool for the study of mortality risk and its spatial variations.

## Background

Colorectal cancer (CRC) is a major health problem in Western countries. According to recent studies, 2.2 million cases and 1.1 million deaths are expected from this cause by the year 2030, worldwide [1]. CRC is the second most common cancer in women, after breast cancer, and in men, after prostate cancer. Among both sexes, it is the third most common cancer worldwide [2], and the second cause of cancer mortality in Europe [3–6].

The incidence and mortality of CRC present marked geographical differences; it is found more frequently in developed regions. In the last 20 years, the mortality due to CRC has declined significantly in northern and western Europe, while it has increased in most countries in southern, central and eastern Europe [4]. In this context, Spain is among the middle-ranking countries in Europe. The incidence of CRC and the resulting mortality are expected to continue rising, to more than 37,000 episodes and 18,000 deaths in 2020 in Spain [1, 7]. Epidemiological studies in Spain reflect a gradual increase in mortality in recent years [8–10], as well as in incidence, probably because the disease is being detected at an increasingly early stage, with the use of new diagnostic techniques [11].

The distribution of mortality patterns of CRC in Spain presents some variability among regions (in Spain, termed Autonomous Communities) and over time. López Abente et al. [10] studied periods from 1989 to 2008, to determine the mortality patterns of the most commonly-occurring cancers in Spain. In relation to CRC, the main characteristics found were an increase in mortality in cities in Catalonia and in the province of León during the first 5 years among men, and in these locations during the first 10 years among women.

Standardised mortality ratios (SMRs) can be used to detect and resolve problems of hospital treatment quality, and to facilitate inter-hospital comparisons based on case studies. First described by Jarman et al. in this context [12], SMRs are based on the creation of a specific model for each hospital, adjusted for the characteristics of its patients, in such a way that the mortality recorded can be compared with that expected from logistic regression calculations that allow the circumstances to be adjusted by an indirect method. In the UK, researchers have obtained a positive correlation between the SMRs for individual hospitals and those for the corresponding geographic area [13]. However, to our knowledge no studies have been conducted in Spain in which the SMR method is applied to geographic regions (rather than specific hospitals) as a means of detecting heterogeneities and of determining spatial patterns in this country.

Although current data and long-term projections are available for CRC mortality in Spain [8], very little is known about in-hospital mortality due to this disease, and no current data are available for spatial and temporal distributions based on hospital data.

The aim of the present study, therefore, is to detect patterns of geographic heterogeneity for the excess risk of death due to CRC during hospitalisation episodes, estimated by the SMR method, in Spain during the period 2008–2014 and, secondarily, to describe the temporal evolution of these SMRs for each region, during the same period.

## Methods

### Study design and data source

This study focuses on hospitals belonging to the Spanish National Health System, a decentralised structure of 17 autonomous health systems. The study cohort constructed contains all the hospitalisation episodes that took place in Spain during the period 2008–2014. The information source used was the Minimum Basic Data Set (MBDS) at hospital discharge for the above period, facilitated by the Health Information Institute of the Spanish Ministry of Health, Consumer Affairs and Social Welfare (IIS-MSC, Spanish initials). All patients diagnosed with CRC (as the primary or secondary diagnosis) were included.

The selection criterion applied for the inclusion of cases was that of the International Classification of Diseases, 9th edition, Clinical Modification [14]. All episodes of hospitalisation according to ICD codes 153 (colon cancer) and 154 (cancer of the rectum and rectosigmoid junction) were analysed in patients aged 20 years or more. In consequence, the unit of analysis was the hospitalisation episode, and not the individual patient. The data were then compiled by health regions to calculate the corresponding SMRs.

Data on CRC mortality in Spain for the reference population during the study period were obtained from the IIS-MSC. These mortality rates were then adjusted for age and sex, by the direct method and taking the world population as a reference value.

### Study variables

The main dependent variable analysed was mortality due to any cause during a hospitalisation episode (in patients admitted for CRC, codes 153 and 154 ICD-9-CM). In addition, sociodemographic variables (age, sex, region) and clinical variables (number of diagnoses at discharge -NDD- and number of procedures at discharge -NPD- performed prior to discharge) were analysed. NDD is a proxy variable of comorbidities and complications, and NPD is a measure of treatment effort. The variable “level of severity” is obtained from certain characteristics of patients, especially secondary diagnoses and procedures performed; this variable behaves similarly to the Charlson index but is stratified into four levels of severity: minor (1), moderate (2), greater (3) and extreme (4). This adjustment variable is expressly provided in the IIS-MSC database.

Also analysed were the type of admission – urgent or programmed – and management variables (total and preoperative stay, type of hospital and readmission). Readmission was defined as a second or subsequent admission taking place within 30 days of the first and with respect to the same diagnosis-related group.

### SMR calculation

In this study, SMRs were used to compare mortality rates by region for each of the study years (cross-sectional study axis) and also per year for each region (longitudinal study axis). The SMRs were calculated as the ratio (× 100) between the observed and expected cases. The former were obtained directly from the MBDS and the expected cases were calculated using an indirect adjustment procedure based on binary logistic regression, in which mortality was the dependent variable and patient age, length of hospital stay, type of admission, sex and level of disease severity were the predictor variables. The corresponding 95% confidence intervals were calculated according to Byar’s approximation [15]. For the cross-sectional study axis, the expected values of the binary logistic regression were calculated for each year (seven models, one per year, in which the regions were compared within each year). The expected cases of CRC for each year were quantified as the sum of these values in each region. For the longitudinal axis, the values predicted by binary logistic regression were obtained for each region (excluding the autonomous cities of Ceuta and Melilla, hence 17 models, describing the temporal evolution of each region separately). The sum of the predicted values for each year quantified the expected cases for the region in question. The discriminative capacity of the logistic models was evaluated according to the area under the curve (C-statistic) and was considered acceptable when values higher than 0.70 were obtained.

The SMRs were calculated following the method described by Foster et al. [16] and applied in 2010 in the Netherlands, but considering hospitalisation episodes, not individual patients. For this reason, only in-hospital mortality was considered, rather than the method adopted in other studies in which mortality up to 1 month after discharge was taken into account. Also following the above method, no adjustment was made for the involvement of palliative care units [17].

### Cross-sectional analysis

In the cross-sectional analysis, funnel plots were obtained as a graphic representation of each year of the study period [18, 19], with confidence limits of 95% (action limit) and 99.8% (alarm limit) as control lines, corresponding to ±2 and ± 3 standard deviations, respectively. The central axis of the diagram represents a risk ratio of 1 (SMR 100%), that is, the situation in which the observed and predicted cases coincide. The data between the control lines are considered to be in the range of “common variations”, i.e. due to normal data variability. Beyond these limits, the data lie within an area of “variation due to special causes” and require investigation to determine why, in addition to random phenomena, there was a deviation from the mean of this magnitude [16].

In our analysis, the regions where the control lines (action and alarm limits) were exceeded are represented on the corresponding funnel plots. For each region and year, the SMR was calculated, together with the respective 95% confidence interval, by Byar’s approximation.

### Longitudinal analysis

For the longitudinal analysis and temporal trends, the SMRs were tabulated by region and by year, with the respective 95% confidence intervals (Byar’s approximation). The C-statistic ranges were determined for the models corresponding to each region. The regions where in one or more years the deaths from CRC exceeded the predicted values by 15% or more were then identified.

Population mortality rates were calculated and adjusted to the universal population by the direct method, in order to obtain a reference framework. The average rate for the period was calculated and mapped according to two visible categories (above and below the mean).

## Results

During the study period 258,927 episodes of hospitalisation were analysed. The average hospital stay was 13.16 (SD 12.07) days and 60.6% of the patients were male. The average number of diagnoses made prior to discharge, taken as a proxy variable of comorbidity, was 6.67 (SD 3.44). On average, 3.61 (SD 3.02) total (surgical and clinical) procedures were performed prior to discharge. In 50.2% of the episodes considered, a surgical procedure was performed on admission. By type of admission, 55.1% were programmed. 15.7% of the patients were in readmission, and 10.6% died during hospitalisation.

Among patients aged over 20 years, the number of hospital admissions for CRC during the study period rose from 34,111 in 2008 (crude rate 73.90 × 100,000 inhabitants) to 38,591 in 2014 (crude rate 82.51 × 100,000 inhabitants) (+ 13.13%). Among male patients, admissions rose from 20,569 to 23,453 (+ 14.02%) and among females, from 13,541 to 15,137 (+ 11.79%). The values for average length of hospital stay and of preoperative stay and for the prevalence of readmissions declined during the study period. Conversely, patient age, comorbidities and procedural complexity tended to increase (Table 1).

**Table 1:** Descriptive statistics of the sample

		2008	2009	2010	2011	2012	2013	2014
Total hospitalisations: n (rate × 100,000 inhabitants)		34,111 (73.9)	36,437 (77.95)	35,953 (76.46)	37,944 (80.41)	37,542 (79.43)	38,349 (81.37)	38,591 (82.51)
Sex (Male) n (%)		20,569 (60.30)	21,984 (60.30)	21,654 (60.2)	23,039 (60.70)	22,756 (60.60)	23,471 (61.20)	23,453 (60.80)
Age (mean ± sd)		70.07 ± 11.95	70.18 ± 11.94	70.21 ± 12.02	70.32 ± 12.02	70.36 ± 12.06	70.38 ± 12.08	70.47 ± 12.13
Days hospital stay (mean ± sd)		14.84 ± 13.25	14.27 ± 13.11	13.64 ± 12.25	12.96 ± 12.08	12.56 ± 11.60	12.26 ± 11.08	11.84 ± 10.88
Days preoperative stay (mean ± sd)		4.55 ± 7.40	4.19 ± 7.36	3.73 ± 6.67	3.16 ± 5.98	2.85 ± 5.57	2.7 ± 5.31	2.35 ± 4.75
Surgery n (%)		17,691 (51.90)	18,830 (51.70)	18,925 (52.6)	19,012 (50.10)	18,686 (49.80)	17,903 (46.70)	18,989 (49,20)
NDD (mean ± sd)		5.94 ± 3.17	6.19 ± 3.24	6.41 ± 3.27	6.7 ± 3.45	6.94 ± 3.50	7.13 ± 3.56	7,24 ± 3.64
NPD (mean ± sd)		3.5 ± 2.87	3.57 ± 2.92	3.52 ± 2.93	3.61 ± 2.98	3.64 ± 3.06	3.71 ± 3.15	3,72 ± 3.17
Urgent admission (%)		15,710 (46.10)	16,796 (46.10)	16,521 (46.00)	17,154 (45.20)	16,551 (44.10)	16,684 (43.50)	16,561 (43,00)
Readmissions (%)		5596 (16.40)	5891 (16.20)	5685 (15.80)	5984 (15.80)	5775 (15.40)	5866 (15.30)	5785 (15.00)
Mortality (%)		3826 (11.20)	3966 (10.90)	3875 (10.80)	3995 (10.50)	3920 (10.40)	3917 (10.20)	3950 (10.20)

*NDD: Number of diagnoses prior to discharge; NPD: Number of procedures prior to discharge*

**Fig. 1:**
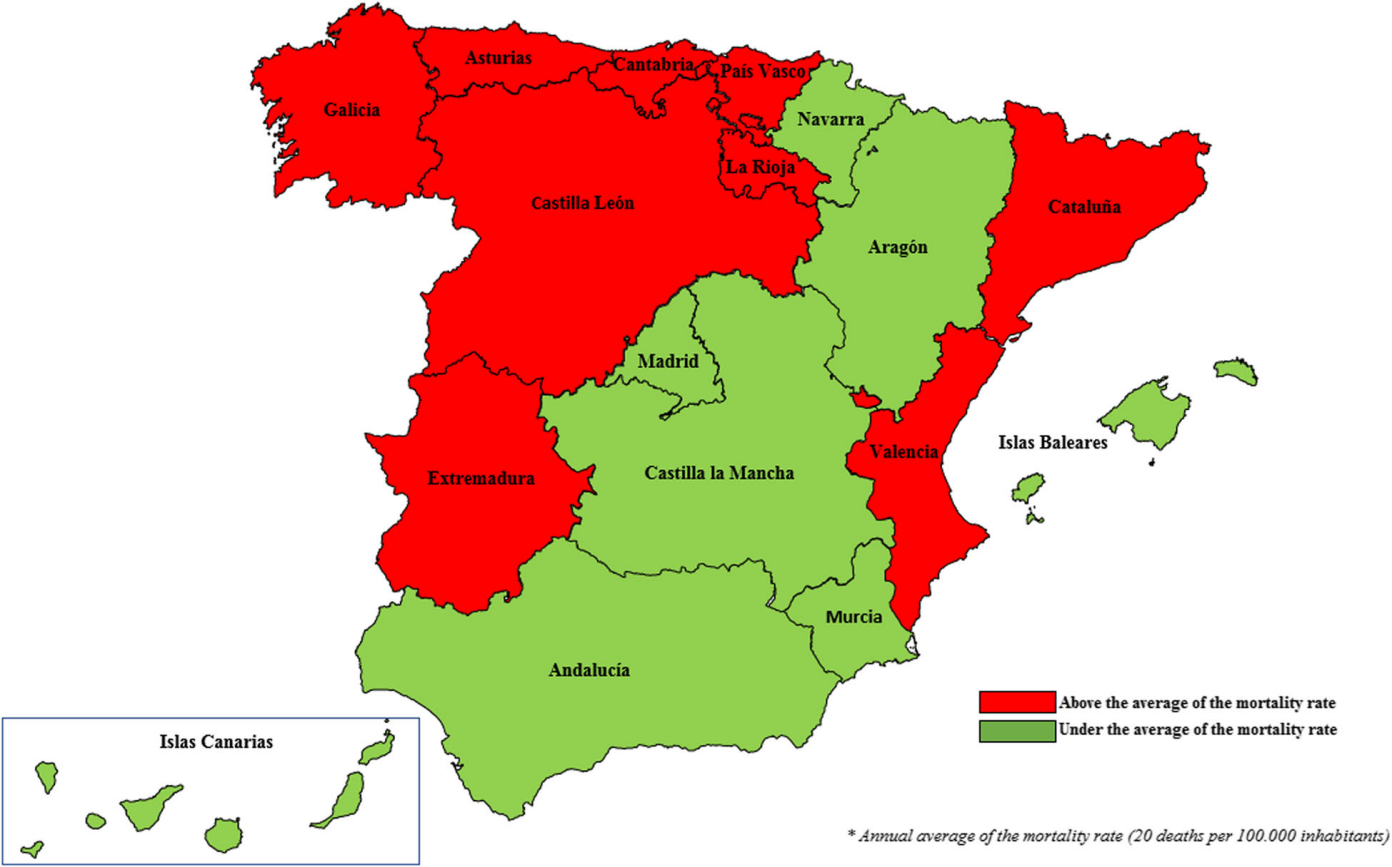
Mortality rates adjusted by the direct method. Period 2008–2014 (Population data. Source: INE)

Nationally, and during the study period, the annual mean death rate from CRC was 20.0 per 100,000 inhabitants. Rates above this average were recorded in Galicia, Asturias, Cantabria, País Vasco, La Rioja, Castilla León, Extremadura, Valencia and Cataluña, in a pattern that reflected considerable geographic heterogeneity, with notably higher rates of mortality in the north and northwest of the country (Table 2 and Fig. 1).

**Table 2:** Mortality from CRC per 100,000 inhabitants in Spain, period 2008–2014 (Population data. Source: INE)

REGION	2008	2009	2010	2011	2012	2013	2014	Mean
Andalucía	19.70	19.94	19.72	19.89	20.63	19.72	19.55	19.88
Aragón	17.37	19.14	18.81	20.69	21.66	19.23	19.63	19.50
Asturias	21.70	23.82	21.40	25.15	23.16	22.40	22.74	22.91
Islas Baleares	20.59	19.74	18.92	20.14	20.07	17.81	18.58	19.41
País Vasco	21.40	20.45	21.06	20.71	20.60	19.91	20.29	20.63
Islas Canarias	18.51	18.62	19.66	19.32	19.49	18.63	17.87	18.87
Cantabria	16.13	19.78	21.89	21.48	21.78	21.22	21.57	20.55
Castilla León	21.36	21.86	23.13	22.70	21.31	20.97	20.58	21.70
Castilla La Mancha	17.48	16.39	17.30	19.43	19.46	18.64	18.31	18.14
Cataluña	20.13	20.57	20.54	20.53	20.91	20.42	19.32	20.35
Extremadura	18.49	21.41	21.58	19.77	22.97	25.09	21.05	21.48
Galicia	19.78	19.97	21.58	22.31	21.50	21.36	19.45	20.85
Madrid	18.77	18.40	18.53	17.94	18.56	17.73	17.53	18.21
Murcia	18.68	18.43	19.64	20.55	18.46	18.21	16.82	18.68
Navarra	19.90	18.42	18.93	20.74	16.29	21.35	21.65	19.61
La Rioja	20.34	20.25	19.40	21.90	21.78	18.68	17.97	20.05
Valencia	20.25	20.75	20.64	20.44	19.82	19.48	18.80	20.03

### Results of the cross-sectional analysis of SMRs (inter-region comparisons, for each year studied)

This analysis identifies the regions where the observed cases of in-hospital death due to CRC exceeded those expected. The SMR was deviated by at least 3 SD (the alarm limit) above its maximum value in one or more years in the following regions: Islas Canarias, Asturias, Valencia, Extremadura, Andalucía and País Vasco. In Islas Canarias the deviation was greater than 3 SD in every year of the study period.

**Fig. 2:**
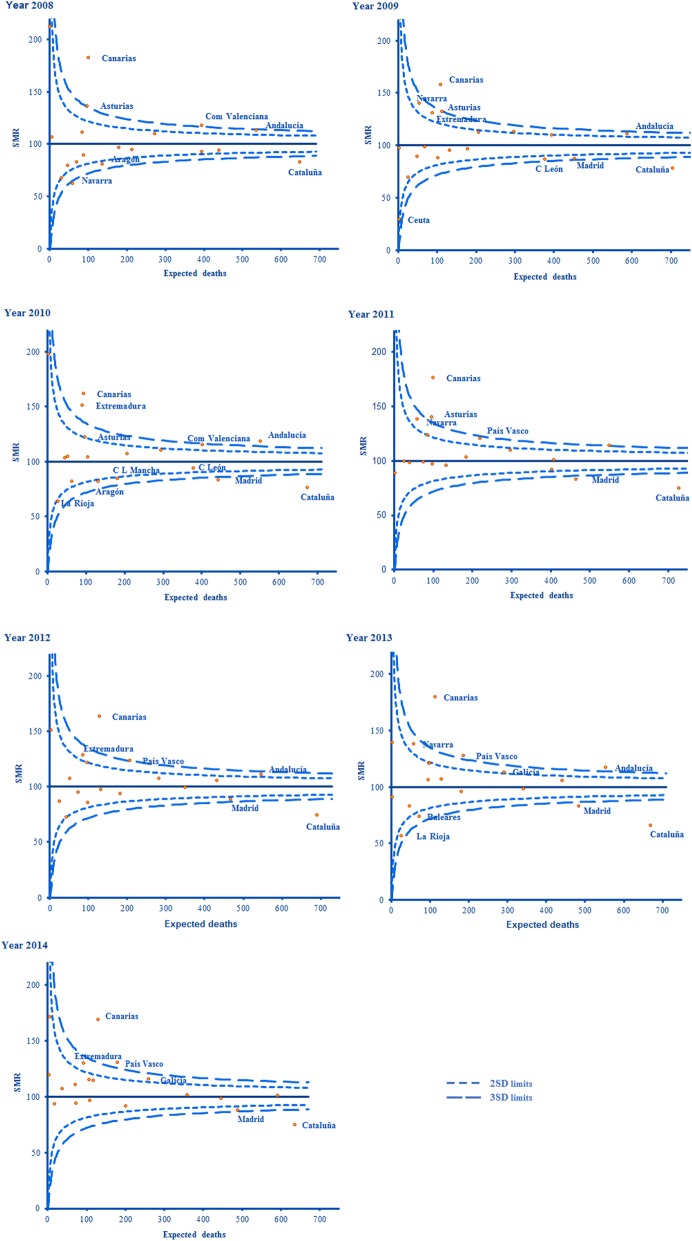
Representation of the SMRs of each health region and year by Funnel Plot diagrams

**Fig. 3:**
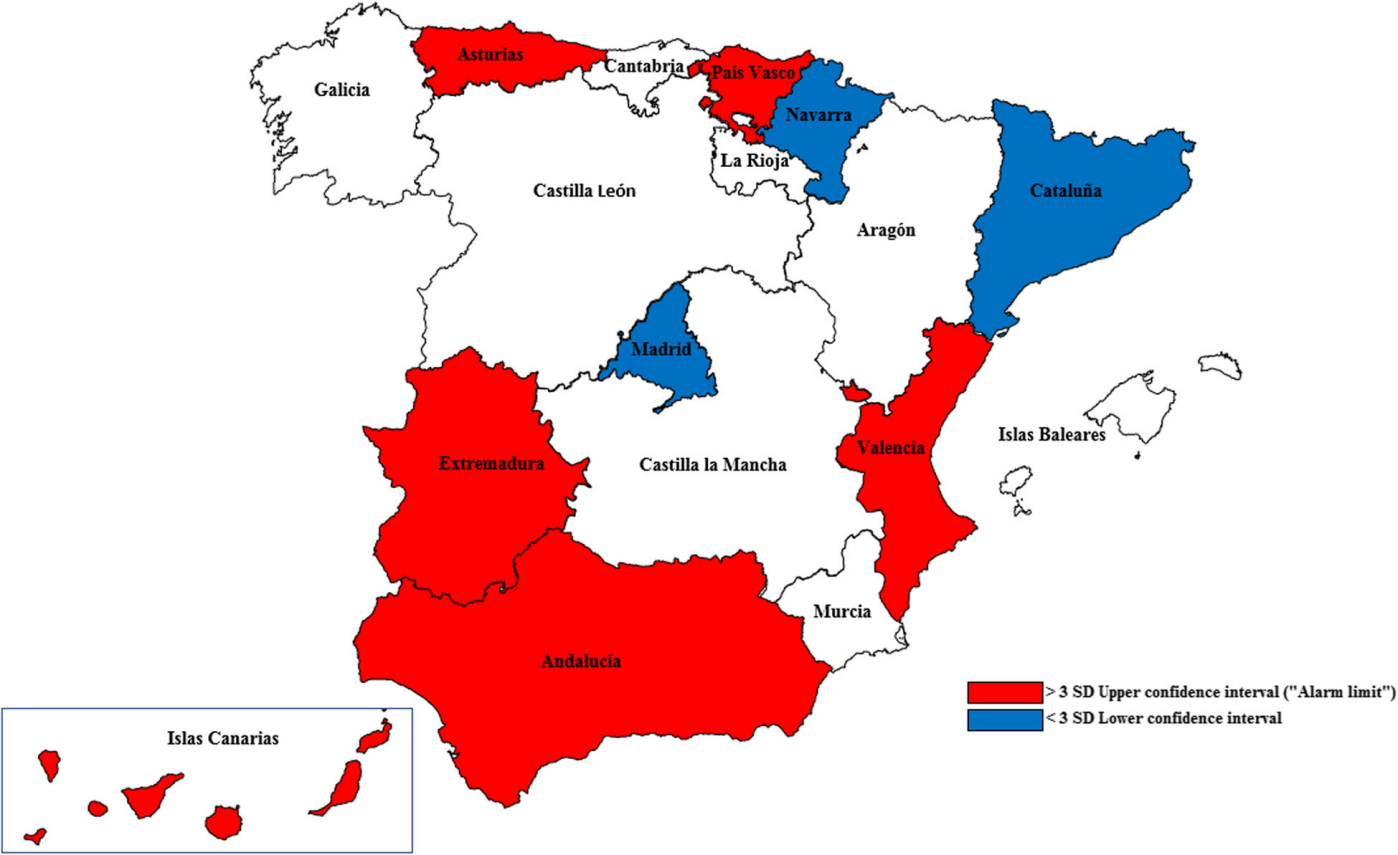
Variations due to special cause (more than 3 SD, upper confidence interval – “alarm limit”-) in the SMRs from CRC

SMRs with deviations up to 3 SD below the confidence interval were detected in at least 1 year in Madrid and Navarra. In Cataluña this happened in all of the years analysed (Figs. 2 and 3).

For each year and region, the discrimination values of the logistic regressions used in the prediction were evaluated. Application of the C-statistic produced areas under the curve ranging from 0.72 to 0.85. (Table 3).

**Table 3:** Comparison of SMRs in each region during the period 2008–2014 (cross-sectional analysis)

REGION	2008	2009	2010	2011	2012	2013	2014
Andalucía	112.37 (103.58–121.71)	110.61 (102.28–119.45)	118.07 (109.19–127.49)	113.96 (105.21–123.24)	110.84 (102.20–120.02)	116.93 (108.10–126.28)	100.56 (92.64–108.97)
Aragón	80.14 (65.92–96.51)	94.65 (78.85–112.70)	81.40 (66.78–98.28)	95.65 (79.85–113.65)	97.33 (81.32–115.58)	106.50 (89.59–125.67)	114.10 (95.66–135.05)
Asturias	136.12 (114.12–161.11)	131.67 (111.38–154.59)	122.12 (101.25–146.03)	129.93 (109.01–153.70)	121.17 (100.30–145.12)	120.61 (99.83–144.43)	114.83 (95.50–136.91)
Islas Baleares	82.34 (62.68–106.21)	98.30 (76.33–124.62)	81.79 (61.26–106.99)	98.46 (77.31–123.61)	94.54 (73.69–119.44)	73.40 (55.13–95.77)	93.73 (72.92–118.62)
País Vasco	94.06 (81.54–107.97)	112.13 (98.19–127.49)	106.97 (93.36–122.00)	120.43 (106.37–135.84)	123.39 (108.74–139.47)	127.27 (111.61–144.51)	129.71 (113.62–147.44)
Islas Canarias	182.10 (156.87–210.24)	157.38 (134.80–182.66)	161.30 (136.75–188.98)	176.26 (151.18–204.31)	163.35 (142.05–186.94)	179.48 (155.75–205.81)	168.43 (146.95–192.16)
Cantabria	79.25 (56.07–108.78)	88.91 (64.59–119.36)	103.17 (76.06–136.79)	97.88 (69.92–133.29)	72.11 (49.31–101.80)	82.80 (59.41–112.33)	106.28 (76.59–143.66)
Castilla Leon	92.16 (82.94–102.14)	86.08 (76.98–95.97)	93.36 (83.90–103.60)	91.53 (82.42–101.36)	98.78 (88.66–109.74)	97.71 (87.52–108.75)	101.20 (91.07–112.15)
Castilla La Mancha	96.38 (82.59–111.81)	96.16 (82.32–111.65)	84.21 (71.43–98.61)	102.77 (88.67–118.47)	93.35 (79.88–108.44)	95.26 (81.59–110.56)	91.35 (78.63–105.54)
Cataluña	82.63 (75.78–89.92)	77.97 (71.59–84.77)	75.77 (69.34–82.63)	74.60 (68.47–81.15)	74.24 (67.96–80.95)	65.28 (59.31–71.69)	74.49 (67.93–81.51)
Extremadura	110.54 (89.33–135.27)	130.62 (108.02–156.55)	150.93 (126.80–178.32)	123.59 (101.28–149.35)	128.14 (105.41–154.32)	120.36 (99.87–143.81)	129.27 (107.35–154.36)
Galicia	109.20 (97.17–122.30)	113.03 (101.28–125.76)	110.12 (98.47–122.76)	109.32 (97.75–121.87)	107.24 (95.51–120.03)	112.93 (101.07–125.80)	114.88 (102.24–128.64)
Madrid	93.47 (84.65–102.96)	87.08 (78.70–96.11)	83.07 (74.81–91.99)	82.56 (74.51–91.24)	88.05 (79.76–96.98)	82.39 (74.51–90.88)	87.27 (79.19–95.95)
Murcia	88.86 (70.35–110.75)	87.72 (70.63–107.71)	103.55 (85.10–124.81)	96.97 (78.55–118.42)	85.19 (67.95–105.48)	105.65 (86.15–128.26)	96.30 (78.84–116.48)
Navarra	61.80 (43.72–84.82)	139.49 (110.26–174.10)	104.42 (78.87–135.60)	137.77 (109.73–170.79)	107.04 (81.07–138.69)	137.78 (109.41–171.25)	110.30 (87.46–137.28)
La Rioja	67.20 (41.58–102.72)	69.46 (41.80–108.48)	63.57 (38.25–99.27)	98.94 (64.61–144.97)	86.66 (54.29–131.21)	56.24 (31.45–92.76)	92.76 (54.95–146.62)
Valencia	117.15 (106.73–128.31)	109.64 (99.56–120.47)	114.80 (104.57–125.76)	100.57 (91.08–110.78)	105.41 (95.96–115.55)	105.57 (96.21–115.59)	98.11 (89.13–107.74)

*SMRs: Standardised mortality ratios. (95% confidence interval, Byar’s approximation)*

*All regressions corresponding to this table provided C-Statistic values within the range 0.72 to 0.85*

Finally, the regions were mapped highlighting those which, in at least 1 year of the study period, presented SMR values above or below the 3 SD alarm limit, and the level between them. This provided a graphic and spatial representation of how the excess risk was distributed in Spain during the study period (Fig. 3).

### Results of the longitudinal analysis of SMRs (outcomes for each region, for the whole study period)

**Fig. 4:**
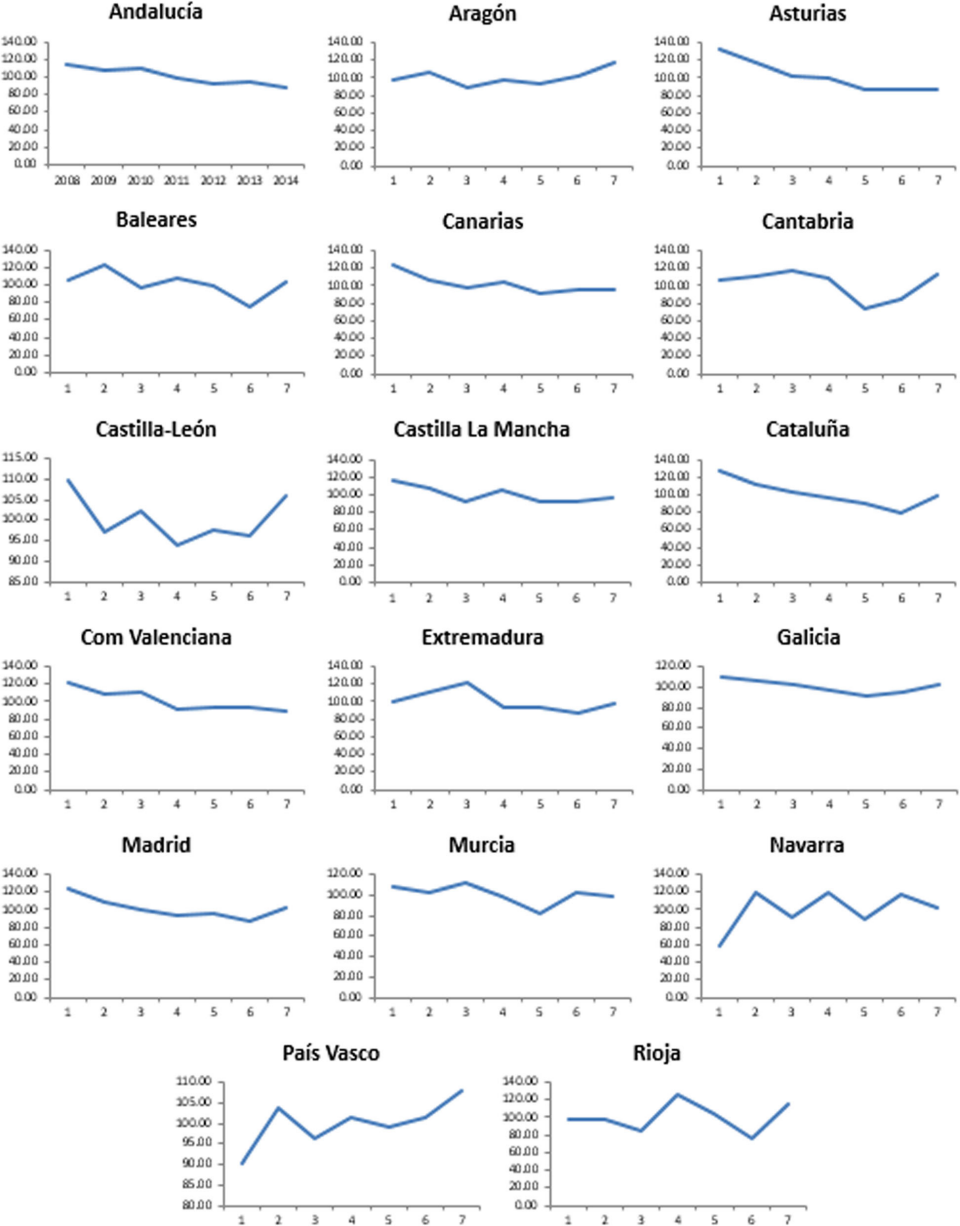
SMRs in each region during the study period (longitudinal analysis)

Analysis of the evolution of the SMR in each region during the study period (Fig. 4) showed that practically every region, at one time or another, presented SMR values > 100, i.e. excess mortality with respect to the expected value. An excess of observed deaths equal to or greater than 15% (SMR ≥115) was detected in Asturias, Islas Baleares, Islas Canarias, Castilla-La Mancha, Cataluña, Valencia, Extremadura, Madrid, Navarra, Aragón, Cantabria and La Rioja. Although many of these cases were isolated peaks in a single year, in Asturias, Navarra and La Rioja the SMR was greater than 115 in at least 2 years. All SMRs were evaluated according to the calibration of their corresponding logistic regression models, producing an area under the curve that was always > 0.72.

## Discussion

The study results obtained illustrate the spatial distribution in Spain of in-hospital mortality due to CRC during hospitalisation episodes, and its evolution during the study period. The present study was undertaken to determine the level of discordance between observed and expected hospital deaths during hospitalisations due to CRC in each of the 17 health regions in Spain. The analysis performed shows first how the SMR behaved for each Community and year and for each Community throughout the study period. In this study, therefore, we present two difficult-to-reconcile but essential approaches: the comparison between different Communities (Table 3) and the evolution over time for each Community (Fig. 4). With the study method employed, the only way to determine the excess risk in space and time simultaneously is to use separate temporal and spatial trend analyses.

In Spain, previous studies have documented inequalities between small geographical areas in cancer mortality, due to non-oncological reasons. These differences are frequently related to socioeconomic inequalities [5, 20].

In our case, the degree of excess risk was calculated by contrasting the observed and expected data using a linear probabilistic model based on logistic regression. This indirect method of standardisation produces smaller standard errors than those obtained by the direct method [16, 21].

We conclude that, during the study period, the geographic pattern of mortality in Spain did not coincide with the excess risk of mortality calculated using the SMR method. This method of risk estimation can be useful for studies of mortality risk and its spatial variations in hospitalisation episodes for CCR.

Before discussing our results, and contrasting them with previous research findings, we highlight the following strengths and limitations of the study method employed.

### Advantages and main limitations

To our knowledge, this is the first application in Spain of the Jarman and Foster method to evaluate the excess mortality in hospitalisation for CRC, with respect to geographic regions and not to individual hospitals. The Jarman-Foster method has been used extensively to analyse the quality of hospital care, to detect deficiencies and to highlight areas for improvement [22]. However, it has not been employed to compare hospital quality levels in small and medium-sized geographic units, such as the 17 regions of Spain, the sole exception to this being the study conducted by McCormick in the UK [13]. In our approach, which is based on hospitalisation episodes and not on individual patients, SMRs provide an appropriate means of comparing mortality rates between regions and with respect to different time periods.

The first limitation of our analysis is that the imbalances observed with respect to the excess of risk only reveal regions that might hypothetically be conflicting, but these findings need to be corroborated by more specific methods. On the other hand, the method described facilitates the automatic detection of possible conflict areas, inexpensively and quickly.

In our opinion, use of the method we describe should not be limited to inter-hospital analysis. However, the application of non-universal surgical techniques, together with the existence of hospital units where only palliative care for CRC is provided, may further increase the variability of the results obtained. This method is subject to some controversy because the denominator for the SMR estimation is obtained from a logistic regression [23]. Another limitation factor, the availability of palliative care units, seems to have most impact on SMRs, while surgical techniques appear to make less difference, as their use tends to be generalised throughout the country [24, 25]. In any case, in Spain there are still few palliative care units and in this regard we believe that the possible bias introduced is minimal [26]; nevertheless, hospital deaths occur, whether or not the patient is admitted to a palliative unit, and therefore we cannot exclude the possibility that this may slightly bias the SMR estimations obtained.

Other limitations to this study concern the source of information used, the MBDS. This is fundamentally a high-quality record, and the volume of episodes presented is such that it may be considered to describe quasi-population structures. However, it does suffer from a certain lack of completeness. Nevertheless, and with specific reference to CRC patients, use of the MBDS has produced good research outcomes, and it is considered to be a suitable data source for cancer registers [27, 28].

The MBDS sometimes presents coding deficits, especially the under-recording of well-known chronic pathologies, which can produce the Jencks bias [29], by which paradoxical behaviour patterns are obtained from certain predictor variables, falsely suggestive of a protective influence. In our study, the effect of this well-known bias is compounded by the difficulty of working with hospitalisation episodes rather than individual patients.

### Case-mix regarding the cases studied

In line with previous research in Spain and elsewhere in Europe [11, 30–32], our results show that mortality from CRC is higher in male than in female patients, in all regions. The prevalence of in-hospital CRC mortality is 10.6% and its distribution presents evident geographic asymmetry. The mortality data obtained differ from those presented in other recent studies and from data published by the Spanish National Institute of Statistics [33, 34]. A possible explanation for this discrepancy is that in-hospital deaths are not fully coincident with deaths during the first surgical admission, but may occur in a subsequent readmission. Moreover, our analysis is based on in-hospital data, which can be expected to vary from those obtained from other population sources.

Etxeberria et al. studied CRC mortality in Spain during the period 1975–2008, using spatial-temporal models of conditional autoregression [30]. These authors observed an increasing risk of mortality in men, in the northern and central parts of Spain, the Mediterranean area and in some southern provinces. Among patients aged 50–69 years, the risk stabilised from 2001, but in those aged over 70 years, the risk continued to increase. A high risk group for CRC was located in the provinces of north-western Spain. Among female patients, the temporal model remained fairly flat throughout the period, although among those aged 50–69 years, the risk increased slightly towards the end of the period. Across the country, the mortality risk clearly decreased from north to south and from west to east among those aged 50–69 years, while among older patients these differences were less apparent. The latter data are consistent with our own findings, especially among male patients, for whom the risk exceeding 3 SD in the geographic areas specified is very similar to the results cited. The official statistics showing adjusted average mortality rates for the period 2008–2014 [34] reveal high mortality rates in the northern, western and southern regions of Spain. Consequently, the overlap between mortality rates and excess risk is only partial and there is no particularly striking agreement between the two scenarios.

### Case-mix with respect to excess risk

According to our study results, the spatial distribution of the excess risk of mortality from CRC is very heterogeneous. Thus, Islas Canarias, Asturias, Valencia, Andalucía, Navarra, Extremadura, País Vasco and Galicia all presented a pattern of moderately high excess mortality (SMR > 2SD) (Fig. 2). Among these regions, Asturias, Islas Canarias, Valencia, Extremadura, Andalucía and País Vasco presented at least one year’s values with an extreme deviation (“alarm limit”, SMR > 3 SD) during the study period (Fig. 3).

These high-risk areas (Fig. 1) only coincide to a certain degree with the population mortality rates for the period in question (Fig. 3). Consequently, while it was possible to determine a set of geographic areas of very high risk (exceeding the alarm limits), in very few regions did the excess risk for hospital mortality coincide with high rates of mortality.

Various factors might be responsible for the non-coincidence of regions with excess risk and those presenting high mortality rates. On the one hand, mortality from CRC could be affected by heterogeneity in the implementation of screening programmes [11, 35] and/or by socioeconomic inequalities [5]. On the other hand, the latter outcome might be associated with environmental, nutritional and even behavioural factors, and probably then with complex interactions between these factors.

The concept of excess risk during hospitalisations can also be viewed in terms of its mathematical formulation. When the numerator is greater than the denominator, the observed value exceeds the predicted one. This is true for any mortality rate or ratio, large or small, and it need not resemble the adjusted mortality rate for the period. Thus, the excess risk is just an indication of how much higher the mortality is in a given area than what would be expected, mathematically.

Recent studies [5, 10, 30] have observed specific spatial-temporal patterns in the distribution of CRC mortality in Spain. These studies conclude that differences in the distribution of resources and the unequal geographic implementation of screening programmes are strongly related to the patterns detected.

The estimates obtained using the direct method revealed above-average rates of mortality for the period 2008–2014 in the northwest and northeast of the country and in the eastern and western corridors to the north of Andalucía. These findings are similar to those of Etxeberría et al.; however, our results differed strongly in that we detected a north-south corridor of low risk, composed of Cantabria, Navarra, Aragón, Madrid, Castilla la Mancha, Murcia and Andalucía.

Comparison of the findings reported by Etxeberría et al. for the period 1975–2008 [30], for the geographic distribution of CRC mortality, with our own results shows that the respective study periods overlap, with our research focusing on more recent years. Nevertheless, there is a marked congruence in the areas of greatest risk for mortality by CRC except in the southwest.

### The pattern of SMRs over time and the evolution of excess risk

Among the geographic units analysed, Aragón, País Vasco and Navarra all presented rising trends of excess mortality risk (Fig. 4). In most of the regions considered, and throughout the study period, there was no congruence between areas with high SMR and those where mortality rates were also high. In our opinion, therefore, there is probably no relationship, either, between the evolution of SMRs over time and the static mortality rate observed in any given region. However, we did not perform a specific test in this regard and so the latter statement constitutes a hypothesis that remains to be considered in future studies of this question.

## Conclusions

In Spain, unlike its neighbours, no previous study has been made of the excess risk of mortality from CRC using the Jarman and Foster method, performed with respect to hospitalisation episodes, rather than patients. Our findings show that in this country, during the period 2008–2014, the geographic distribution of the excess risk of mortality from CRC differed substantially from the official mortality rate. Hence, we observed no clear relationship between the areas that presented higher risk and those where mortality was objectively higher. In consequence, mortality and excess risk do not appear to be strongly associated, and their respective geographic patterns cannot be superimposed. The reasons for this non-coincidence might be found in socioeconomic inequalities and in differences in accessibility to treatment.

We conclude that SMRs, as a measure of excess risk in CRC mortality, constitute a useful tool for studying the risk of mortality, together with the presence of heterogeneous patterns and unequal resources, among the regions of Spain.

We believe it would be feasible to implement a monitoring system applied to the regions rather than individual hospitals. This approach would highlight, in real time, statistical deviations regarding mortality risk and the possible existence of under-provided areas, thus enabling appropriate corrective measures to be taken.

It is of crucial importance to highlight geographic differences that may be a source of inequality in the availability of treatment and/or care, in order to identify areas of quality deficit. Doing so would facilitate the design of short to medium-term strategies to detect problematic regions at an early stage and thus enable the rapid application of corrective measures. Finally, the problem considered may also reflect the presence of variability in clinical practice. This could undoubtedly be detected, as a secondary impact, and then addressed rapidly and efficiently.

## Abbreviations

CRC: Colorectal cancer
ICD: International Classification of Diseases
IIS-MSC: Ministry of Health, Consumer Affairs and Social Welfare
INE: Spanish National Institute of Statistics
MBDS: Minimum Basic Data Set
NDD: Number of diagnoses prior to discharge
NPD: Number of procedures prior to discharge
SD: Standard deviation
SMRs: Standardised mortality ratios
